# Biaxial Orientation of PLA/PBAT/Thermoplastic Cereal Flour Sheets: Structure–Processing–Property Relationships

**DOI:** 10.3390/polym15092068

**Published:** 2023-04-26

**Authors:** Nour Jaouadi, Racha Al-Itry, Abderrahim Maazouz, Khalid Lamnawar

**Affiliations:** 1 Université de Lyon, CNRS, UMR 5223, Ingénierie des Matériaux Polymères, INSA Lyon, Université Claude Bernard Lyon 1, Université Jean Monnet, F-69621 Villeurbanne, France; nour.jaouadi@insa-lyon.fr (N.J.);; 2 Université de Sfax, ENIS, Laboratoire Electrochimie et Environnement LEE, Sfax 3038, Tunisia; 3 NLMK, 1 Rue Bassin de l’industrie, 67016 Strasbourg, France; 4 Hassan II Academy of Science and Technology, Rabat 10100, Morocco

**Keywords:** polylactic acid, thermoplastic cereal flour/starch, PLA/PBAT blends, biaxial deformation

## Abstract

This paper investigates the biaxial stretchability of polylactic acid (PLA)/poly (butylene adipate co-terephthalate) (PBAT)/thermoplastic cereal flour (TCF) ternary blends with a PLA/PBAT ratio close to 60/40 and a constant TCF content. A twin-screw extrusion process was used to gelatinize the starch and devolatilize the water in order to obtain a water-free TCF, which was then blended into a compatibilized or non-compatibilized PLA/PBAT matrix, introduced in the molten state. These blends were subsequently cast into sheets and biaxially drawn using a biaxial laboratory stretcher. The prepared ternary blends were found to present a typical ductile behavior. Scanning electron micrography highlighted dispersion and adhesion properties in the PLA/PBAT/TCF blends, where two different phases were observed. Moreover, the addition of the thermoplastic cereal flour did not significantly affect the biaxial stretchability of the PLA/PBAT blends but was found to lower the maximum stress before breaking. The modification of the interfacial tension between PLA and PBAT with the compatibilizer Joncryl before mixing with TCF had no effect on the durability of the PLA/PBAT/TCF sheet. Still, it slightly increased the maximum of nominal stress before failure.

## 1. Introduction

Stimulated by strong societal, economic and, more particularly, ecological pressure, numerous studies have been carried out to develop blends of biodegradable polymers with good thermo-mechanical properties [1,2,3]. Poly (lactic acid), PLA, biocompatible aliphatic polyester derived from renewable resources, such as corn and starch, is currently considered one of the most promising biodegradable polymers. It is used in many fields of application, including packaging, electronic housing, and automobile interiors, thanks to its mechanical properties [4,5,6]. However, to be able to use it for certain applications requiring specific properties, improvements are still required regarding its thermo-mechanical properties, its resistance to humidity and its processing conditions. A wide variety of binary blends with PLA have been proposed as a technical solution to improve its toughness [7], such as PBST [8], PCL [9], PHB [10], PA11 [11,12], PBAT [13], etc.

Poly (butylene adipate-co-terephthalate), PBAT, is an auspicious aliphatic-aromatic copolyester. Previous work has proven that PLA/PBAT blends can be a suitable solution to each individual polymer’s physical and mechanical drawbacks [14,15] due to the balance of biodegradability and physical properties [16]. Nevertheless, an incompatibility caused by the differences in hydrophilicity of these two polymers leads to poor mechanical properties of the blend. PLA/PBAT blends thus need to be compatibilized in order to strengthen the interface between phases.

The addition of reactive agents can improve the compatibility between PLA and PBAT by modifying the interfacial adhesion [17]. Examples of compatibilizers include dicumyl peroxide (DCP) [18,19], tetrabutyl titanate (TBT, Ti (BuO4)) [20,21], bioxazoline [22], phthalic anhydride [22], acetyl tributyl citrate [23,24], glycidyl methacrylate (GMA) and Joncryl ADR^®^ [17].

The reactive extrusion of polymers has been performed with various amounts of a chain extension agent. The incorporation of, for instance, glycidyl methacrylate (GMA) functions or Joncryl ADR^®^ into PLA/PBAT has led to an improvement in the thermal stability and an increase in the viscosity and molecular weight of PLA [17]. Reactive agents can promote in-situ copolymerization through the free radical reaction using a peroxide initiator. Nishida M. et al. [19] studied the effect of dialkyl peroxide on PLA/PBAT blends. The results showed that the elongation at break and the impact strength were improved when adding dialkyl peroxide with a ratio of PLA: PBAT: dialkyl peroxide at 60:40:1. Dialkyl peroxide was found to improve the ductility of the blends due to a crosslinking reaction. From these findings, peroxide could be used to modify the morphology and mechanical properties of PLA/PBAT blends. In addition, organically modified nanoclay (C20A) has been added to optimize the mechanical properties of PLA [25] and PLA/PBAT blends [26].

Furthermore, compatibilization with cottonseed oil derivatives, i.e., epoxidized (ECSO) and maleinized (MCSO) cottonseed oil, led to a remarkable increase in elongation at the break without compromising other mechanical resistance properties. Neither ECSO nor MCSO has a relevant effect on overall disintegration, but both oils contribute positively to enhancing the thermal stability, as revealed by thermogravimetry. The use of vegetable oil-derived compatibilizers (epoxidized and maleinized vegetable oils) stands out as an environmentally friendly solution to improve the miscibility of polyester-type PLA/PBAT blends with an excellent balance between mechanical and thermal properties [27].

Another limitation of PLA is its high cost. To overcome this, a commonly used strategy is to blend it with thermoplastic starch (TPS), which has many merits, in particular, its renewable origin, its compostability, and its low cost. At low water content, its oxygen barrier properties are comparable to those of conventional barrier polymers such as EVOH or PVDC [28]. The mechanical properties of PLA/TPS blends appear to be largely dependent on the concentration of plasticized starch, its glycerol level and its humidity level.

PLA and TPS are mainly used for packaging, in the agricultural sector, personal hygiene items, and textiles and have widespread use in medical technology. Mixing starch with PLA has the advantage of reducing the cost but, above all, increasing the degradation rate.

The compatibilization strategy is another important issue in producing PLA/TPS blends since PLA and TPS are immiscible polymers, and their mixtures have a heterogeneous morphology in which TPS usually constitutes the dispersed phase in the continuous phase of PLA.

Among the authors who have explored the subject, Martin et al. [29] have shown that the addition of a very small amount of starch to a PLA matrix dramatically lowers the mechanical properties. The impact resistance of 12 kJ/m^2^ for pure PLA drops to 2 kJ/m^2^ with only 25% starch. The same is true for the elongation at break and the elasticity limit.

PLA and TPS have a high interfacial tension in the melt state, leading to very coarse blend structures. In addition, the two materials do not inherently have good interfacial adhesion in the solid state. The use of a compatibilizer is therefore required. The efficiency of the compatibilization largely depends on the diffusion of the reactive component migrating to the interface and to the chain entanglement between the modifier and the pure phases in the interfacial region.

The most studied compatibilizer in the literature for TPS/PLA mixtures is maleic anhydride (MA) [30]. Huneault et al. demonstrated an improvement in ductility through the functionalization of PLA, under the action of peroxide, with MA before mixing with TPS containing more than 36% glycerol. The obtained mixture was homogeneous. This resulted in a finely improved morphology with a reduced dispersed phase. Likewise, the substitution of hydroxyl groups in starch molecules with acetyl groups has been performed to increase starch’s hydrophobicity and thermal stability [31].

Another study investigated the efficacy of dioctyl maleate as a compatibilizer. The results obtained were less satisfactory [32]. Other investigations have shown that adding montmorillonites (MMT) can also lead to an increase in resilience.

Much research has dealt with the chain extender effect (Joncryl ADR 4370S) on TPS and plasticized PLA (p-PLA) films. An improvement in elongation at break was observed for a 70/30 (TPS/p-PLA) film [33].

However, some authors have shown that the elongation at break can be enhanced by incorporating a third flexible biodegradable polymer like polycaprolactone, PCL [34]. PCL can be introduced in the molten process, thus becoming mixed with the PLA. It appears that adding 10 wt % of PCL increases the impact of the resistance and ductility, as reported by Sarazin et al. [34]. Indeed, blending a PLA/TPS binary blend with another flexible polymer could be a useful way to obtain a new kind of material with excellent performance. Poly (butylene adipate-co-terephtalate) (PBAT) is a flexible copolyester that can fully degrade within a few weeks. It could be considered a good candidate for toughening rigid polymers such as TPS/PLA binary blend.

Other studies highlight the improvement of interfacial tension of ternary blends of PLA/TPS/PBAT prepared via a reactive modification [35,36]. Three types of compatibilizers were tested: ethylene diphenyl diisocyanate (MDI), maleic anhydride (MA) and maleic anhydride grafted polyethylene (MA-g-PE). MDI displayed superior compatibilization as opposed to the other two due to it giving rise to improved thermal and mechanical properties.

Chapleau et al. [37] have studied the effect of molecular bi-orientation on the improvement of mechanical in PLA/TPS blends. They have shown increased tensile strength, elastic modulus and elongation at break with the deformation ratio. In this work, we tried to replace the TPS with thermoplastic cereal flour (TCF) to study the effect of the flour on the mechanical properties of PLA/PBAT blends under biaxial simultaneous equi-biaxial deformation. We should note that the studied blends were prepared by Ulice, an industrial project partner. A biaxial laboratory stretcher was used to perform the tests. The influence of the draw ratio at the fixed temperature on the maximum stretching was also developed. Moreover, the impact of the interfacial adhesion between PLA and PBAT polymers on the mechanical behavior of ternary PLA/PBAT/TCF blends will also be investigated.

## 2. Experimental Section

### 2.1. Materials

**Figure 1:**
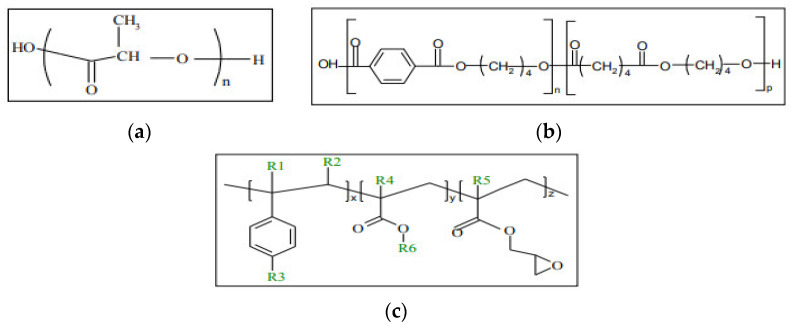
Chemical structure of (**a**) PLA, (**b**) PBAT, and (**c**) general structure of the styrene-acrylic multifunctional oligomeric chain extenders. Where R1–R5 are H, CH3, a higher alkyl group, or combinations of them; R6 is an alkyl group, and x, y and z are each between 1 and 20. Joncryl ADR^®^-4368 [17,39].

The PLA Grade 4032D purchased from Natureworks (Plymouth, MN, USA) has a D-isomer content of approximately 2%, an average molecular weight of 100.000 g/mol (GPC analysis), a glass transition and melting temperatures of approximately 60 °C and 170 °C (DSC analysis), respectively. Concerning P (BA-co-BT) or PBAT, the copolymer was supplied by BASF SE (Ludwigshafen, DE) Grade Ecoflex FBX 7011. The molar fraction of BT and BA units are 44% and 56%, respectively. It exhibits a weight average molecular weight of 40.000 g/mol (GPC analysis), a glass transition temperature and a melting point of −30 °C and 110–120 °C (DSC analysis), respectively. Both polymers are supplied in pellet form. Commercially available Joncryl (BASF (Ludwigshafen, DE), Joncryl ADR^®^–4368) has been used as Chain Extender polyester molecules. It is an epoxy-functional oligomeric acrylic with the following physical characteristics: T°g = 54 °C, EEW (equivalent epoxy weight) = 285 g/mol, Mw = 6800 g/mol, obtained in flake form [38], and the thermoplastic cereal flour (TCF) used is the biolice 50T was provided by Limagrain, France; it is a bioplastic, compound of plasticized cereal flour and biodegradable polyesters. The rate of flour used in the blend is 24% and 9% of glycerol, which was chosen as a flour plasticizer. The chemical structures of PLA, PBAT and Joncryl ADR^®^ are shown in Figure 1.

### 2.2. Blend Compounding

All pellets (PLA/PBAT/TCF) were received from Ulice_Limagrain, France. They were prepared by a twin-screw extruder as follows: The first half of the extruder was used to prepare the thermoplastic cereal flour (TCF). The initial starch content was 24 wt %, and the glycerol content was 9 wt %. The water was removed by vacuum before mid-extruder to obtain TCF with minimal residual water.

The PLA/PBAT blends were incorporated in the molten state (at 190 °C) at midextruder through a single screw extruder side-feed. To modify the interface, 0.5 and 0.9 wt % of Joncryl ADR-4368 were added to the PLA and PBAT blends before being mixed with TCF. It is believed that the residual epoxy groups initially present in Joncryl ADR-4368 were able to react with the hydroxyl groups from TCF and glycerol.

### 2.3. Biaxial Stretching of Cast Sheets

The films were extruded in two steps; firstly, the pure/modified PLA and PBAT polymers and their blends were mixed by a co-rotating twin screw extruder (was provided by Thermo Electron PolyLab System Rheocord RC400P (Waltham, MA, USA) with screw diameter of 16 mm). The profile temperature was set at 140, 190, 190, 180 and 180 °C. The residence time was about 2 min. More details about their rheological, thermal, mechanical, and morphological properties are presented in previous papers of Al-Itry et al. [17,39,40]. It should be pointed out that the incorporation of Joncryl makes film processing more stable.

The pellets were prepared as described above. Once received, they were dried in a vacuum oven at 80 °C for 12h before processing. Cast films were extruded with a 30-mm single-screw extruder and quenched at 40 °C (chill-roll temperature). The melt temperature was set at 180 °C; the temperature profile was set at 177, 200, 200, 190, 200, and 190 °C from the feed zone to the die with a screw speed of 90 rpm, and the residence time was maintained at 3 min. The amorphous obtained films had a final thickness of about 500 µm.

The square test specimens were cut from the uniform central region of the extruded films. The uniformity of the cross-shaped specimen thickness was verified and validated. The description of the simultaneous biaxial stretching method has been presented in previous work [38]. In this study, the biaxial testing experiments were conducted at 75 °C. The stretching rates were chosen to be 0.2 and 0.4 s^−1^, corresponding to 4 and 10 mm/s, respectively. The thickness of the films was thus reduced significantly (~50 µm).

Table 1 lists the various studied samples. The blends had varying PLA/PBAT ratios and a constant TCF content. Various amounts of Joncryl ADR^®^ of 0 and 0.5% wt were used for TCF+PLA, PBAT and their blends. It should be pointed out that the addition of Joncryl ADR^®^ (0 and 0.5% wt) increased the consistency of the film quality and made the film extrusion process more stable. Without Joncryl ADR^®^, fractures and defects were formed, and the film broke quickly.

## 3. Results and Discussions

### 3.1. Preliminary Study

**Figure 2:**
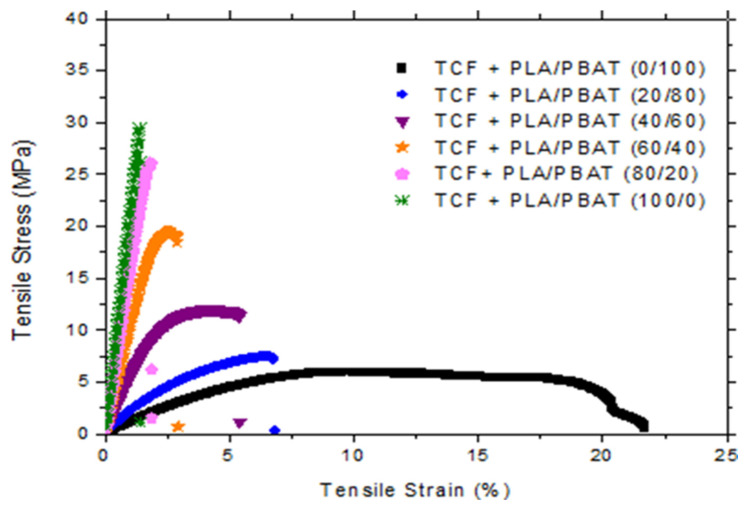
Typical tensile stress–strain curves for cast PLA/PBAT/TCF sheets with a constant “TCF” content.

The main objective of this preliminary study was to characterize the mechanical behavior of different ternary PLA/PBAT/TCF blends upon uniaxial deformation. The obtained results help us to choose the optimal PLA/PBAT ratio for the biaxial stretching experiments. The elastic modulus, the yield stress and the elongation at break were determined. They are summarized in Table 2 and plotted in Figure 2.

Al-Itry et al. [17] demonstrated that the elastic modulus of pure PLA was about 1350 MPa. The tensile properties of the PLA/TCF blend (100/0 PLA/PBAT ratio into PLA/PBAT/TCF blends) showed an increase in the elastic modulus of PLA by the addition of TCF. It varied from 1350 MPa for PLA pure to 2103 MPa for PLA/TCF. This is to be expected with the addition of a soft component.

Concerning the PLA/PBAT/TCF blends, a high elastic modulus (going from 96.5 MPa to 2103 MPa) and a high yield stress (from 7.4 MPa to 29 MPa) were obtained with the addition of PLA. Therefore, the results suggested that PLA could impart strength and rigidity to the bends due to its relatively high yield stress (75 MPa) and its high elastic modulus, as reported in the literature for PLA [26].

However, it should be pointed out that the incorporation of PBAT into a PLA/TCF matrix allowed a transition from a brittle to a ductile behavior which was highlighted by the increase of the elongation at break (going from 1.2% to 14.5%).

**Figure 3:**
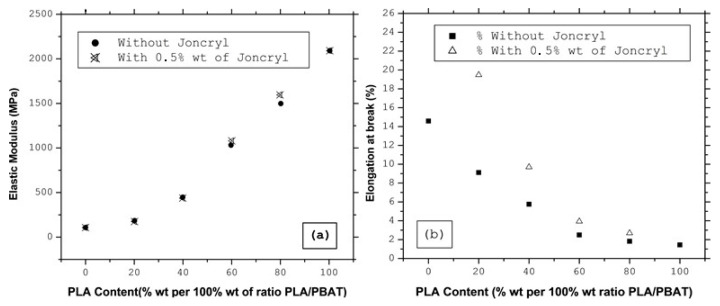
Evolution of the (**a**) elastic modulus and (**b**) elongation at break for varying PLA/PBAT/TCF blends with and without Joncryl.

With the addition of 0.5 % wt of Joncryl (which acted as a compatibilizer for the PLA/PBAT blends [17,38]) into PLA/PBAT before mixing with TCF, the elastic modulus of the ternary blends was nearly unaffected by the interfacial modification, as shown in Figure 3. This did not come as a surprise, considering that PLA/PBAT/Joncryl had reacted for only a few seconds with TCF. This meant that the Joncryl did not have sufficient time to react with the hydroxyl groups of the TCF. However, the effect of the good interfacial adhesion of PLA/PBAT in the PLA/PBAT/TCF blends was more significant when measuring the elongation at break, which was enhanced for PLA/PBAT ratios of 20/80, 40/60, and 60/40.

**Figure 4:**
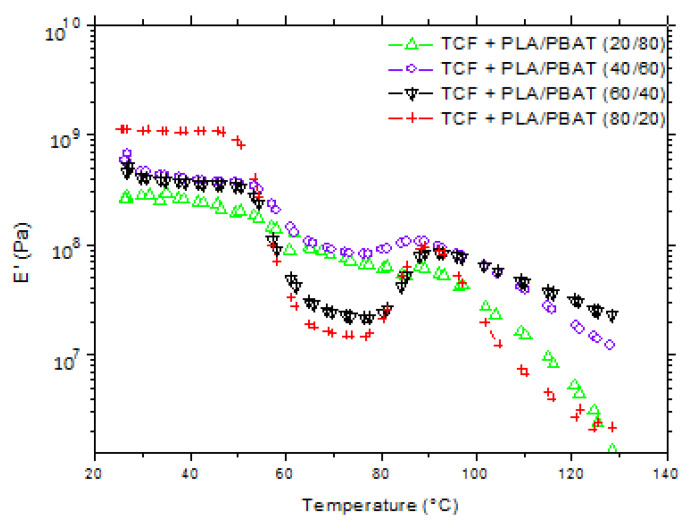
Storage modulus (E’) versus temperature for various TCF/polyester blends.

It should be noted that Joncryl has the role of a processing aid. Figure 4 displays plots of the storage modulus (E’) of TCF versus temperature.

DMTA studies revealed that all blends presented a decrease in E’ over the glass transition temperature of PLA from 50 to 65 °C, suggesting that the material was becoming less elastic. The addition of Joncryl did not significantly affect the principal relaxation of PLA, demonstrating that Joncryl had no plasticization effect on the films.

As the PBAT content increased and the PLA content was lowered, the value of E’ decreased, indicating that the blends with less PBAT were more elastic. These results corroborated those of the uniaxial tensile strength. The processing temperature for the biaxial experiments was estimated thanks to DMTA measurements. It was determined between the *α*-relaxation temperature and the onset temperature for the cold crystallization, representative of PLA. According to the obtained curves, a temperature around 75 °C was chosen for the biaxial orientation experiments.

Based on the obtained results, we decided to use the abovementioned preparation method (cf. blend compounding) to prepare ternary samples with a PLA/PBAT ratio of 60/40 for the biaxial orientation experiments.

**Figure 5:**
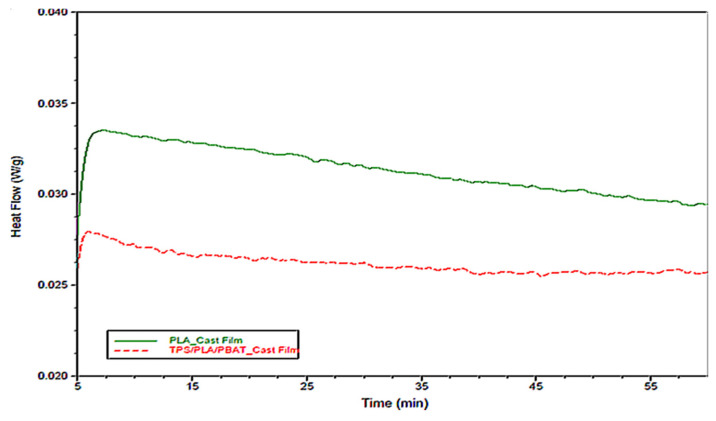
Isothermal crystallization for PLA and PLA/PBAT/TCF (PLA/PBAT = 60/40) ternary blends at 75 °C for 60 min.

Moreover, as shown in Figure 5, an investigation of the isothermal behavior of the studied blends at 75 °C confirmed that no induced thermal crystallization could develop in the performed tests since the time scale of the test (preheating + stretching) was approximately 10 min, even though the starch could play the role of the nucleating agent for PLA in PLA/PBAT/TCF [35,36,41].

### 3.2. Properties of the Bi-Axially Stretched Films

The various compositions are listed in Table 3. Cast PLA/PBAT/TCF films were produced with both 0.5 wt % and 0.9 wt % (an excess) of Joncryl with the expectation of a possible reaction between the three components, which led to an enhancement of the deformation of the film.

**Figure 6:**
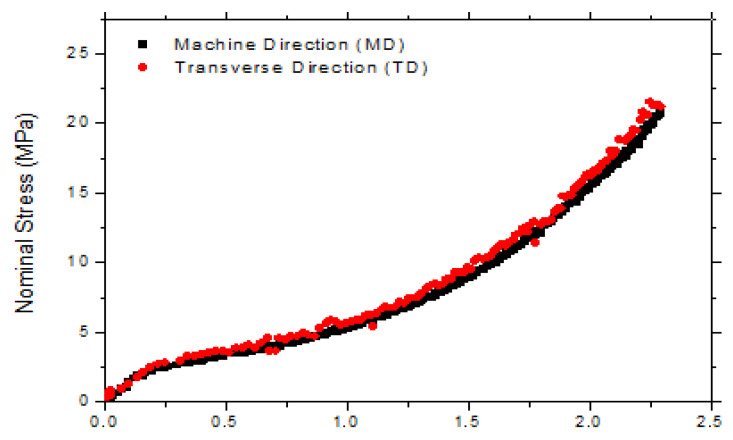
Stress–strain curves of ternary PLA/PBAT/TCF blends in both the machine and transverse directions.

**Figure 7:**
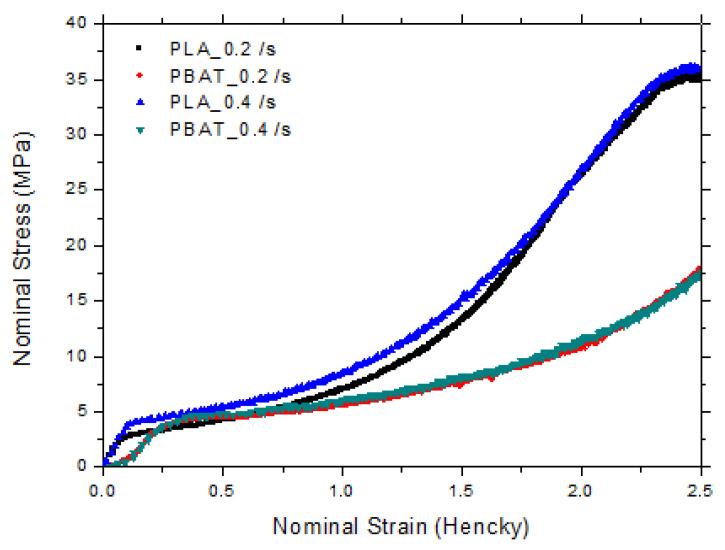
Stress–strain curves in machine direction for PLA and PBAT at two strain rates (0.2 and 0.4 s^−1^).

**Figure 8:**
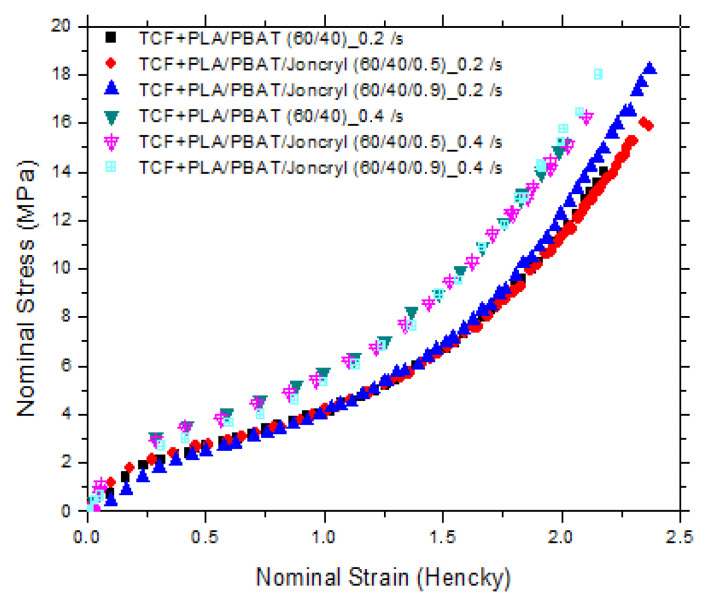
Stress–strain curves in the machine direction for PLA/PBAT/TCF at two strain rates (0.2 and 0.4 s^−1^).

The nominal stress vs. nominal strain curves at 75 °C is shown in Figure 6. We should point out that the stresses in both the machine and transverse directions were similar, which is representative of the initial isotropy of the cast sheets. For further analysis, only the data for the machine direction are shown in the graphs at 75 °C for two different strain rates (0.2 and 0.4 s^−1^), as can be seen in Figure 7 and Figure 8.

Table 4 summarizes the obtained results of the uniaxial strain hardening parameter (SHP) (or optimum draw ratio) for studied samples. It was shown that PLA and PBAT could be axially stretched to a maximum before failure close to 1.5 and 2.25 Hencky at 75 °C, respectively, regardless of the strain rate. The mechanical properties of biaxial simultaneous drawing for PLA/PBAT blends have been discussed in our previous work [38].

It was observed that the stretchability of these blends depended on the PLA/PBAT ratio and that it was improved for high amounts of PBAT.

The interfacial modification of the blends by the incorporation of a compatibilizer agent, Joncryl ADR, led to an earlier strain-hardening. This phenomenon was related to the fact that Joncryl also reacted as a nucleating agent for PLA, thus allowing an increase in the crystallization kinetics; the physical crosslinking of the macromolecular network by the crystallites, thereby preventing the chain relaxation.

For the unmodified PLA/PBAT/TCF, typical ductile characteristics of the plastics were observed. It was found that the addition of TCF did not significantly affect the stretchability of PLA/PBAT while lowering the maximum stress before the break from ~25 MPa for PLA/PBAT [38] to ~15 MPa for PLA/PBAT/TCF. Since TCF has a greater affinity with PBAT compared to PLA [38], we concluded that upon drawing, the PLA matrix exhibited elongated voids around the granular PBAT/TCF particles. Clearly, PLA debonded during drawing, leaving a cavitated structure at Hencky strains of 0.2 and 0.4 s^−1^.

**Figure 9:**
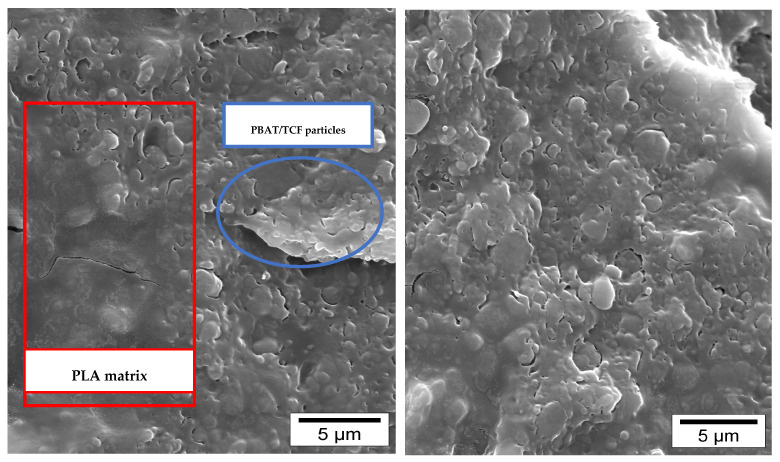
SEM micrograph of PLA/PBAT/TCF with 0.9 % wt of Joncryl.

The incorporation of Joncryl ADR into the PLA/PBAT blends before the mixing with TCF did not affect the stretchability of the PLA/PBAT/TCF film but slightly increased the maximum stress before failure, which was probably related to the improvement of the interfacial adhesion between PLA and PBAT. A SEM micrograph highlighted the poor adhesion in the PLA/PBAT/TCF blends where two different phases corresponding to PLA and PBAT/TCF were observed, as shown in Figure 9.

## 4. Conclusions

The properties and processability of TCF/polyesters were investigated. It was found that the elastic modulus of PLA decreased with the addition of TCF. For PLA/PBAT/TCF blends, the results suggested that PLA could impart strength and rigidity to the bends. It should also be pointed out that the incorporation of PBAT into the PLA/TCF matrix rendered possible the transition from a brittle to ductile behavior. Moreover, with the addition of 0.5 % wt Joncryl into the PLA/PBAT/TCF blends, the elastic modulus was practically unaffected by the interfacial modification but became more significant when measuring the elongation at break. It was also found to be enhanced for PLA/PBAT ratios of about 20/80, 40/60, and 60/40.

During biaxial stretching experiments at 75 °C of the unmodified PLA/PBAT/TCF, typical ductile characteristics of the plastics were seen. The addition of TCF did not significantly affect the stretchability of PLA/PBAT while lowering the maximum stress before the break. The incorporation of Joncryl into PLA/PBAT blends prior to the mixing with TCF had no effect on the stretchability of the PLA/PBAT/TCF film but gave rise to a slight increase of the maximum stress before failure. SEM highlighted the poor adhesion in the PLA/PBAT/TCF blends, where two separate phases were observed.

While Joncryl is a generally accepted compatibilizer for PLA/PBAT blends, it is important to gain a better understanding of the role of TCF and the effect of its plasticization degree on the final properties. Hence, the obtained nanostructured morphology should be further tailored to improve the resulting mechanical properties.

## Figures and Tables

**Table 1:** Composition of samples with varying PLA/PBAT ratios for the preliminary study.

(a) Samples	% wt Joncryl in the Blend	% wt TCF in the Blend	PLA/PBAT Ratio in the Blend
TCF + PLA/PBAT (0/100)	0	33%	0/100
TCF + PLA/PBAT (20/80)	0	33%	20/80
TCF + PLA/PBAT (40/60)	0	33%	40/60
TCF + PLA/PBAT (60/40)	0	33%	60/40
TCF + PLA/PBAT (80/20)	0	33%	80/20
TCF + PLA/PBAT (100/0)	0	33%	100/0
**(b) Samples**	**% wt Joncryl in the Blend**	**% wt TCF in the Blend**	**PLA/PBAT Ratio in the Blend**
TCF + PLA/PBAT (20/80)	0.5	33%	20/80
TCF + PLA/PBAT (40/60)	0.5	33%	40/60
TCF + PLA/PBAT (60/40)	0.5	33%	60/40
TCF + PLA/PBAT (80/20)	0.5	33%	80/20

**Table 2:** Elastic modulus, yield stress, and elongation at break values for the various prepared samples.

PLA/PBAT Ratio into PLA/PBAT/TCF Blends	Elastic Modulus (MPa)	Yield Stress (MPa)	Elongation at Break (%)
0/100	96.5 ± 6.8	7.4 ± 1.1	14.5 ± 4
20/80	184 ± 24	8 ± 1	9 ± 2
40/60	452 ± 52.4	11.5 ± 0.75	5.7 ± 1.2
60/40	1029 ± 47	19 ± 1.35	2.4 ± 0.4
80/20	1508 ± 97.5	25 ± 3.8	1.8 ± 0.25
100/0	2103 ± 79	29 ± 3	1.2 ± 0.2

**Table 3:** Composition of different samples used for biaxial stretching experiments.

Samples	Composition
PLA	100 % wt PLA
PBAT	100 % wt PBAT
TCF+PLA/PBAT (60/40)	33 % wt TCF in the blend + PLA/PBAT (60/40)
TCF+PLA/PBAT/Joncryl (60/40/0.5)	33 % wt TCF in the blend+ PLA/PBAT (60/40) + 0.5 % wt Joncryl
TCF+PLA/PBAT/Joncryl (60/40/0.9)	33 % wt TCF in the blend+ PLA/PBAT (60/40) + 0.9 % wt Joncryl

**Table 4:** Maximum stress before break and strain hardening parameter values for the prepared samples during biaxial stretching experiments at Hencky strains of 0.2 and 0.4s^−1^.

Samples (=0.2 s^−1^)	Maximum Stress before Break (MPa)	Uniaxial Strain Hardening Parameter (SHP) or Strain Limit ^(*Hencky*)^	Samples (= 0.4 s^−1^)	Maximum Stress before Break (MPa)	Uniaxial Strain Hardening Parameter (SHP) or Strain Limit ^(*Hencky*)^
PLA	35	1.5	PLA	35	1.7
PBAT	18	2.25	PBAT	18	2.25
TCF + PLA/PBAT (60/40)	14	1.7	TCF + PLA/PBAT (60/40)	15	1.4
TCF + PLA/PBAT/Joncryl (60/40/0.5)	16	1.75	TCF+PLA/PBAT/Joncryl (60/40/0.5)	17	1.4
TCF + PLA/PBAT/Joncryl (60/40/0.9)	19	1.65	TCF + PLA/PBAT/Joncryl (60/40/0.9)	19	1.4

## Data Availability

Not applicable.
